# Quick and reliable method for retina dissociation and separation of rod photoreceptor perikarya from adult mice

**DOI:** 10.1016/j.mex.2015.01.002

**Published:** 2015-01-15

**Authors:** Yana Feodorova, Mirja Koch, Sebastian Bultman, Stylianos Michalakis, Irina Solovei

**Affiliations:** a Department of Biology II, Center for Integrated Protein Science Munich (CIPSM), Ludwig Maximilians University Munich, Grosshadernerstrasse 2, 82152 Planegg-Martinsried, Germany; b Department of Medical Biology, Medical University-Plovdiv, Blvd. Vasil Aprilov 15A, 4000 Plovdiv, Bulgaria; c Center for Integrated Protein Science Munich CIPSM at the Department of Pharmacy – Center for Drug Research, Ludwig-Maximilians-Universität München, Butenandtstr. 5-13, 81377 München, Germany

**Keywords:** Sorting of mouse rod photoreceptorsn, Mouse retina, Retina isolation, Papain dissociation, FACS, Rod cell perikarya

## Abstract

A pure and abundant population of adult rod perikarya can be exploited in different studies concerning nuclear functions such as gene expression analyses which aim at elucidating the relationship between cell type and disease [1]. Sorting is based either on specific cell-surface markers or fluorescently labeled reporter proteins. Here, we describe a simple and reliable method for separation of rod photoreceptor perikarya without the use of staining procedures or transgenic mice. This method is limited, however, to sorting rod photoreceptors from adult mouse retina. Mature rods possess an inverted nuclear architecture which is determined by the optical functions of these nuclei [2]. The high backscatter of heterochromatin in the core of the nucleus can be used as a selection criterion for FAC-sorting by forward and sideward scatter.

• The procedure for retina dissociation using the Papain Dissociation System (Wothington Biochemical Corporation) was optimized.
• An easy to follow step-by-step protocol for retina dissociation was devised.
• Rod perikarya were FAC-sorted by forward and sideward scatter based solely on the high backscatter of heterochromatin in their nuclei.

## Graphical abstract

**Figure:**
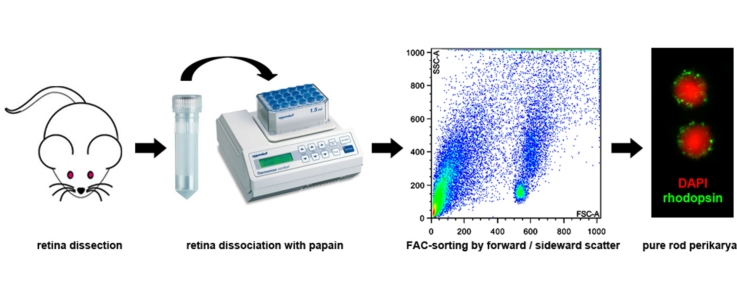

## Method

We describe a quick and reliable method for retina dissociation and separation of rod photoreceptor perikarya from adult mice. The described method is based on FAC-sorting [3] and is applicable only to rod photoreceptors of adult mice (elder than 2 months). The reason for this is that in nuclei of terminally differentiated rod photoreceptors, heterochromatin is compacted in a single dense core with strong light scattering properties [2]. Such unusual nuclear organization of mature rods allows successful sorting of their perikarya without using rod-specific fluorescence.

## Method details

### Animals

ICR/CD1 mice (retired breeders) (Charles River Laboratories, Wilmington, MA, USA) were used in the experiments. All procedures were approved by the Animal Ethics Committee at the University of Munich and were carried out in accordance with German laws and the European Union Directive 2010/63/EU.

### Materials

#### Reagents

• Phosphate buffered saline (PBS), pH 7.4 – prepared from 20× PBS stock (1× PBS: 140 mM NaCl, 2.7 mM KCl, 6.5 mM Na_2_HPO_4_, 1.5 mM KH_2_PO_4_);
• Papain Dissociation System – Worthington Biochemical Corporation (Lakewood, NJ, USA);
• 10% BSA (w/v) (Sigma–Aldrich, St. Louis, MO, USA) in PBS;
• Poly-l-lysine, mol wt 150,000–300,000 (Sigma–Aldrich) – stock solution (10 mg/ml in dH_2_O) is stored frozen at −20 °C; working solution (1 mg/ml in dH_2_O) is discarded after use;
• 4% formaldehyde (w/v) in PBS, pH 7.3 – freshly prepared from paraformaldehyde powder (Fisher Scientific, Waltham, MA, USA) by heating up to 60 °C; PFA has to cool down to RT before usage;
• DAPI (4,6-diamidino-2-phenylindole) (Sigma–Aldrich) diluted to 500 μg/ml in dH_2_O and stored as aliquots at −20 °C; working solution is 0.05 μg/ml in PBS;
• PBST is PBS with 0.2% Tween 20 (v/v) (Merck, Darmstadt, Germany);
• VECTASHIELD mounting medium – Vector Laboratories (Burlingame, CA, USA);
• Blocking solution is PBST with 4% BSA (w/v).

#### Primary antibodies

• Mouse-anti-H4K8acetyl (clone 72A9) was kindly provided by Hiroshi Kimura (Tokyo Institute of Technology), working dilution 1:100;
• Rabbit-anti-H4K20me3 (ab9053) – Abcam (Cambridge, UK), working dilution 1:100;
• Mouse-anti-Rhodopsin (ab3267) – Abcam, working dilution 1:500.

#### Secondary antibodies

• Anti-mouse-Alexa555 (A31570) – Invitrogen (Carlsbad, CA, USA), working dilution 1:500;
• Anti-mouse-Alexa488 (A21202) – Invitrogen, working dilution 1:500;
• Anti-rabbit-DyLight549 (711-505-152) – Jackson ImmunoResearch (West Grove, PA, USA), working dilution 1:500.

#### Instruments

• Dumont #7 curved forceps – Fine Science Tools (Heidelberg, Germany);
• Neubauer chamber – Celeromics (Grenoble, France);
• Microscopic slides, coverslips;
• Transparent nail polish.

#### Equipment

• Thermomixer – Eppendorf (Hamburg, Germany);
• FACS Aria II – Becton Dickinson (Franklin Lakes, NJ, USA);
• Phase contrast microscope equipped with 10× and 20× objectives;
• Epifluorescence microscope equipped with corresponding filters and 63× objective;
• Leica TCS SP5 confocal microscope – Leica (Wetzlar, Germany).

### Microscopy

Single optical sections or stacks of optical sections were collected using a Leica TCS SP5 confocal microscope equipped with Plan Apo 63×/1.4 NA oil immersion objective and lasers with excitation lines 405, 488, and 561 nm. Dedicated plug-ins – StackGroom (zShift corrector and 3channels)–in the ImageJ program were used to compensate for axial chromatic shift between fluorochromes in confocal stacks, to create RGB stacks/images, and to arrange them into galleries [4], [5].

### Experimental procedures

#### Preparation of stock solutions (in the cell culture hood)

- Add 32 ml Earle’s balanced salt solution (EBSS) to Vial 4 (ovomucoid). When everything is thoroughly dissolved, aliquot the solution into 2 ml screw cap tubes, labeled as “tube 4” and store them at +4 °C.
- Add 5 ml EBSS to Vial 2 (papain) and incubate at 37 °C until the solution becomes clear (approx. 10 min).
- Add 500 μl EBSS to Vial 3 (DNase) and gently invert the vial several times to dissolve the DNase; avoid vigorous mixing.
- Add 250 μl of DNase to Vial 2 (papain).
- Aliquot the papain solution into 250 μl aliquots in 2 ml screw cap tubes, labeled as “tube 1”, and store the tubes at −20 °C.
- Aliquot the DNase solution into 15 μl aliquots in 1.5 ml screw cap tubes, labeled as “tube 2”, and store the tubes at −20 °C.
- Prepare 1.5 ml tubes which contain 130 μl EBSS and 15 μl ovomucoid, label them as “tube 3”, and store the tubes at +4 °C.

#### Retina isolation

Mice were killed by cervical dislocation according to a protocol approved by the Animal Ethic Committee of the University of Munich. Retinas were isolated immediately after death. For this, the eye ball was displaced forward by placing a curved Dumont forceps around the posterior part. The cornea was cut using a sharp blade or scalpel, and the retina was squeezed through the cut together with residual pigment epithelium and lens by applying gentle pressure with the Dumont forceps. Dissected retina was placed in cooled PBS, freed from non-retinal tissue using the forceps and immediately transferred to the digestion tube (see below).

#### Papain dissociation of the retina (for 2–4 retinas)

For the retina dissociation we used the Papain Dissociation System and followed the manufacturer’s instructions with small modifications.

- Take tube 1 out of the freezer, let it thaw, slightly open the cap of the tube and equilibrate the solution in the cell culture incubator with 5% CO_2_ for 15–30 min. Tubes should be equilibrated before the retina isolation step.
- Transfer the isolated retinas into tube 1.
- Incubate the retinas in the papain solution for approx. 60 min in a shaker at 37 °C at 700 rpm.

N.B. The dissociation of the tissue in the papain solution should be observed regularly to prevent very harsh disruption of the tissue and improve cell yield.

- Take tube 2 out of the freezer, let it thaw and add the content of tube 3 to it.
- 10–15 min before papain incubation is finished, equilibrate tube 2 and tube 4 in the cell culture incubator with 5% CO_2_ with slightly opened lids.
- After papain incubation, use a 1 ml micropipette (blue tip) and pipet the solution with the partially digested retinas three times up and down. Afterwards, transfer the contents to tube 2.
- Using a long glass Pasteur pipette with a rubber ball, perform mechanical dissociation by pipetting approx. 10 times up and down.

N.B. Pipetting has to be stopped when tissue pieces are no longer visible in the solution.

- Add 250 μl of the ovomucoid solution in tube 4 to tube 2. Mix all the components by pipetting up and down several times using a glass Pasteur pipette. The tube is now ready for FAC-sorting.

The total number of retinal cells derived from papain dissociation was determined manually using a Neubauer chamber. On average, we obtained 13 × 10^6^ cells from 4 mouse retinas.

#### Rod perikarya FAC-sorting

**Fig. 1:**
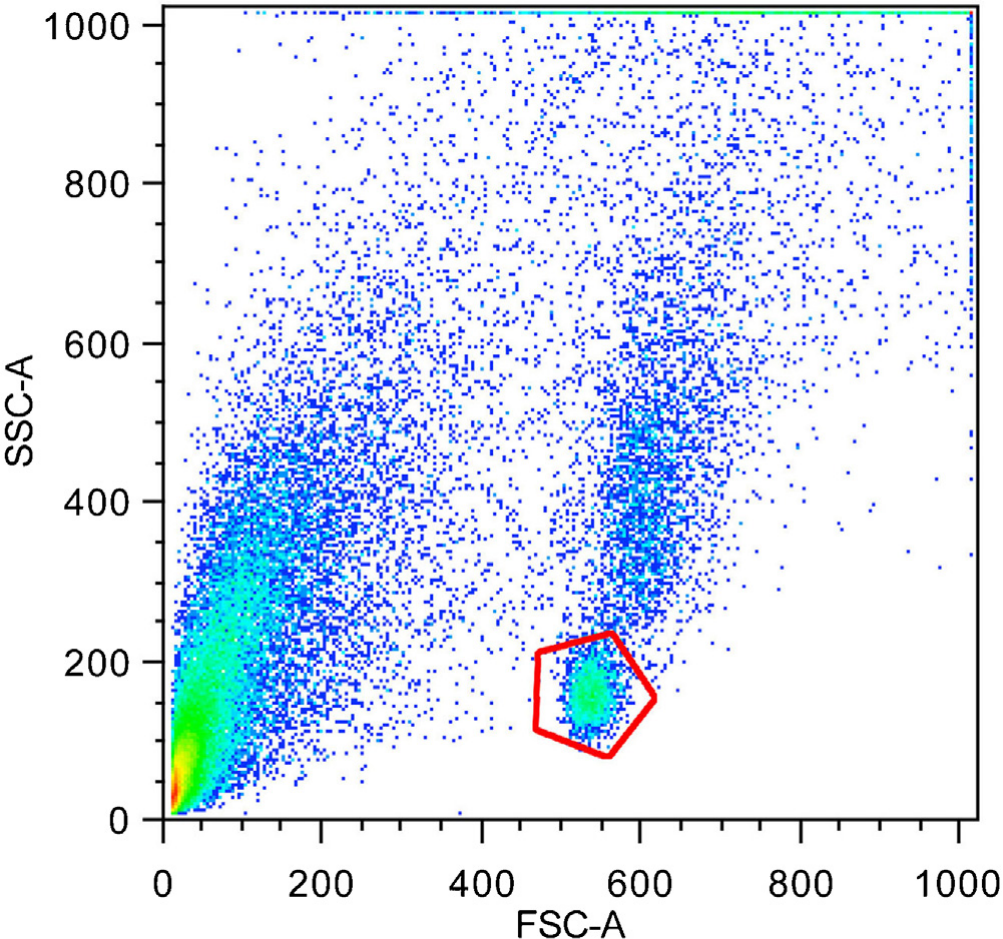
Analysis of isolated retinal cells by flow cytometry. A distinct subpopulation can be observed (red hexagon) which corresponds to rod perikarya.

The resulting suspension was used for cell sorting with FACS Aria II based on size and low internal complexity. Sorting was performed using a 70-micron nozzle. For particle detection standard forward (FSC) and sideward scatter (SSC) settings were used. A distinct subpopulation with low FSC and SSC could be observed. Sorting of this subpopulation yielded pure rod perikarya (Fig. 1).

#### Concentrating the cell suspension

FAC-sorting of rods derived from 4 retinas of CD1 mice yielded 1.5–2.5 × 10^6^ cells in a volume varying between 4 and 10 ml. For some applications, however, cells have to be concentrated in a certain volume or even pelleted. Concentrating rod perikarya by centrifugation is the critical step at which most of the material loss occurred. The reasons for this loss are most probably (i) a very small weight of rod perikarya and (ii) residual electric charge which rod perikarya acquired during FAC-sorting. The concentration problem could be partially solved by mixing of isolated perikarya with 5% BSA.

- Mix the resultant FAC-sorted cell suspension with an equal volume of 10% BSA to achieve BSA final concentration of 5%.
- Centrifuge for 10 min at 400 × *g* (4 °C) and carefully remove the supernatant or leave a small volume inside. Manipulation of the cells from this step on depends on the nature of the downstream experiment.

### Method validation by microscopic control

**Fig. 2:**
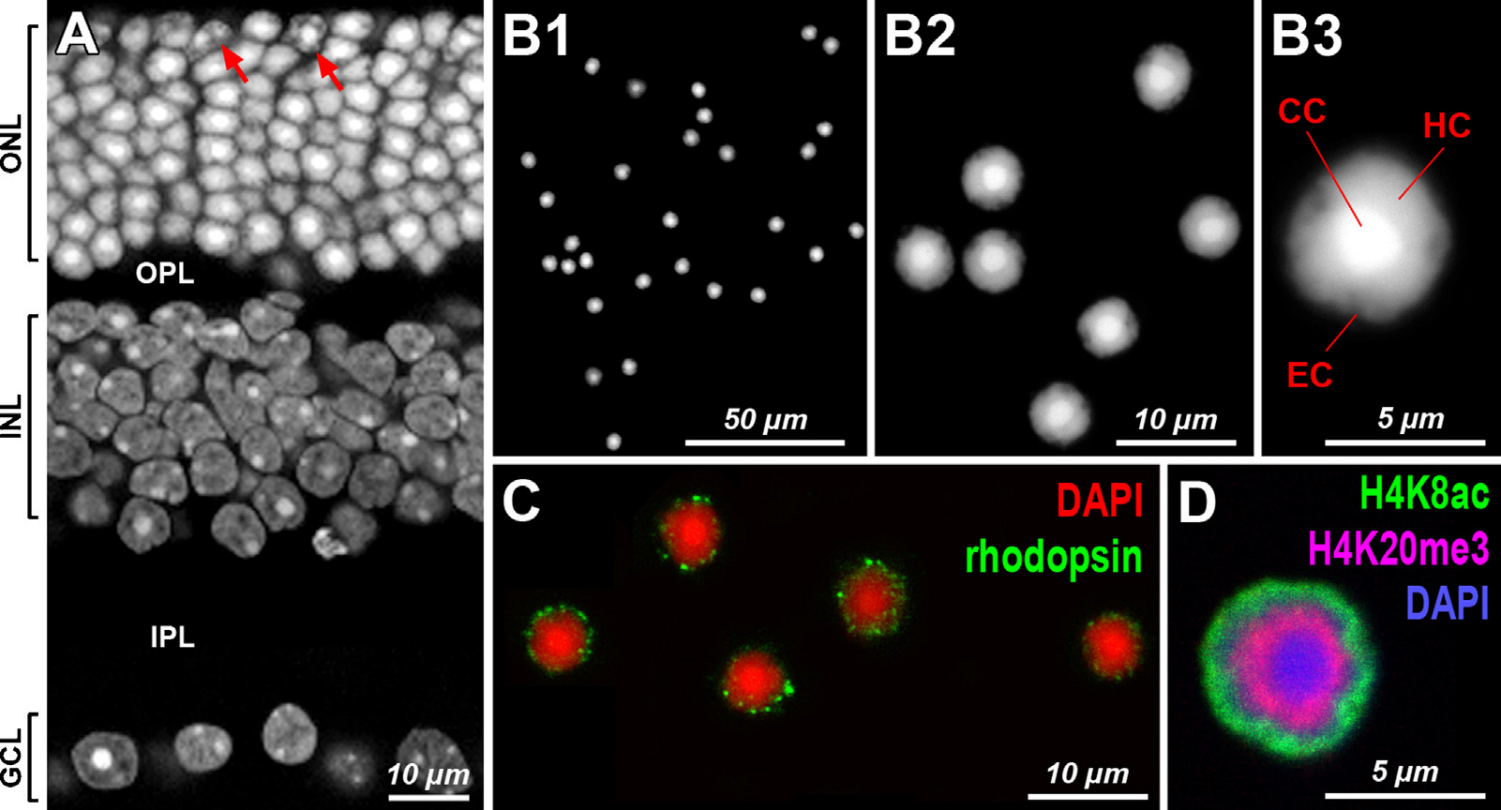
Mouse retina and stainings of rod photoreceptor perikarya obtained by FAC-sorting. (A) Cryosection of adult mouse retina after DAPI staining. Note the bright staining of rod nuclei in the ONL (outer nuclear layer). INL, inner nuclear layer; GCL, ganglion cell layer; OPL, outer plexiform layer; IPL, inner plexiform layer; arrows point at the cone nuclei. (B) DAPI staining of FAC-sorted rod perikarya. (C) Anti-rhodopsin antibody and (D) antibodies against histone modifications applied to FAC-sorted rods. Note that there are three zones of chromatin in rod nuclei stained with DAPI: the brightly stained single chromocenter (CC) is surrounded by less brightly stained heterochromatin (HC) and an outer shell of very weakly stained euchromatin (EC). All images are single optical sections.

Effectiveness of cell dissociation, rod purity after sorting and rod perikarya preservation can be easily controlled microscopically at every step using either phase contrast or fluorescence microscopy. For the phase contrast microscopy, mix a few microliters of the cell suspension with approx. 50 μl of PBS, place it on a microscopic slide under a coverslip, and immediately study under phase contrast microscope equipped with 20× objective (not shown). More detailed information about sorted rods can be obtained after staining nuclei with DAPI. This requires a robust attachment of cells to a microscopic slide which is achieved by a short incubation of cells on slides coated by polylysine. Mouse rod nuclei have a very characteristic morphology and DAPI staining is sufficient for their identification and estimation of their concentration and purity. First of all, rod nuclei are the smallest nuclei in the retina (approx. 5 μm in diameter). Second, rod nuclei possess a very regular concentric arrangement of chromatin classes differentially stained by DAPI: (1) the most centrally positioned chromocenter, consisting of AT-rich major satellite repeat, is very brightly stained; (2) the central chromocenter is surrounded by a shell of less stained heterochromatin; (3) and the most peripheral rod nuclear layer consists of weakly stained GC-rich euchromatin (Fig. 2A and B).

For robust identification of the sorted rod perikarya, one can also use immunostaining of rhodopsin. Since rhodopsin molecules are not very abundant in rod perikarya, the anti-rhodopsin antibody marks sparsely the adult cell bodies (Fig. 2C). For better differentiation of concentric chromatin layers in rod nuclei, one can use markers of eu- and heterochromatin. Thus, the peripheral euchromatin shell in rod nuclei can be highlighted by antibodies against histone modifications characteristic of transcriptionally active chromatin, such as H3K4me3, H3K36me3, and a number of acetylated modifications, e.g., H3K27ac, H4K5ac, H4K8ac (Fig. 2D), H4K12ac, H4K16ac, etc. The heterochromatin shell(s), in contrast, can be stained using antibodies against histone modifications characteristic of heterochromatin, such as H3K9me2,3, H4K20me3 (Fig. 2D), H3K56me3, etc. [6], [7].

Importantly, among FAC-sorted photoreceptors, we have never observed cones which comprise approx. 3% of all mouse photoreceptors. Despite the fact that cones have on average 2–3 large chromocenters, their nuclear architecture is conventional (Fig. 2A) with heterochromatic rims at the nuclear periphery and around the nucleoli. Apparently, cone nuclei, as well as nuclei of neurons from the inner nuclear and ganglion cell layers, have light scattering properties different from those of rods, which allows their separation from rod perikarya.

#### DAPI staining

- A small area, e.g., 18 × 18 mm, is marked by a diamond pencil on the back of a microscopic slide.
- 100 μl of 1 mg/ml polylysine are loaded on the marked area and incubated for 15 min, washed with ddH_2_O, and air-dried. Polylysine coated slides are usually prepared fresh and can be stored for few days at +4 °C.
- 100 μl of the perikarya suspension are loaded to the same marked slide area and incubated for 15 min at room temperature (RT).
- Slides are briefly rinsed in PBS, fixed with 4% formaldehyde for 10 min at RT, and washed with PBS 3 × 5 min at RT.
- Nuclei are counterstained with 0.05 μg/ml DAPI for 5 min at RT.
- Cells are mounted under a coverslip in Vectashield mounting medium and sealed with nail polish.

#### Immunostaining of isolated perikarya

- The first four steps are the same as indicated above for the DAPI staining.
- The antibodies are diluted in blocking solution, loaded on the microscopic slide area with attached cells under a coverslip, and incubated for 45 min.
- Washings after incubations with primary and secondary antibodies are done with PBST, 3 × 5 min.
- DAPI (0.05 μg/ml) for nuclear counterstaining is added to the secondary antibody
- Cells are mounted under a coverslip in Vectashield mounting medium and sealed with nail polish.

## Additional information

### Background

Most studies describing sorting of photoreceptor cells aim mainly at obtaining photoreceptor precursors for transplantation into adult retina affected by degenerative diseases [8], [9], [10], [11]. Trypsin is usually used for dissociation of the retinal tissue but papain has also been utilized as a more efficient and less destructive substance [9], [11]. In our experiment we used the Papain Dissociation System (Worthington Biochemical Corporation) but made some changes to the manufacturer's protocol in order to ease the procedure and improve the quality of the obtained material.

A huge number of donor cells is crucial for transplantation. For this reason all of the above described studies are based on cell surface marker-dependent sorting methods which lead to significant enrichment of donor cells before transplantation [12]. Several such markers have been described [12], [13], [14] but the most widely used one is the glycosylphosphatidylinositol (GPI)-anchored cell surface molecule ecto-5’-nucleotidase (CD73). It has been shown that in the retina of 4-day-old mice it is specifically expressed by rod photoreceptors [14]. Its expression continues after terminal differentiation in rod but not in cone photoreceptor cells [14]. Thus, staining with anti-CD73 antibody represents a possible way to separate rod cells from retina of adult mice.

The advent of powerful high throuput methods (e.g., RNA-seq, proteomics and ChiP-seq) now allows for a more detailed genomic and epigenomic analysis of physiological and pathophysiological processes of the retina. To elucidate cell type specific functions proper methods for isolation of specific retinal cell subtypes (e.g., rods) at high purity and quantity are needed.

Here, we describe a novel and straightforward approach for the isolation of a pure and abundant population of adult mouse rod perikarya. Rods of nocturnal retinas possess a specific inverted pattern of nuclear architecture, where heterochromatin is located in the nuclear center, whereas euchromatin lines the nuclear border. The inverted pattern is established during terminal differentiation of photoreceptors in nocturnal mammals [2] and caused by the absence of two universal tethers of peripheral heterochromatin, lamin B receptor and lamin A/C [15]. In mice, at birth (P0–P7) rod nuclei have a conventional organization with peripheral heterochromatin; later on chromatin arrangement is gradually reorganized into the inverted pattern; the complete inversion is achieved in rods by the 8–10th week of postnatal development [2]. The specific architecture of the rod nucleus results in a specific FSC/SSC pattern during FACS acquisition and enabled us to separate this cell population from the rest of the retinal cells without any staining for specific surface markers.

## Advantages of the method

- Retina dissociation is achieved in 1 h.
- Cell yield is relatively high (up to 2 × 10^7^ cells from 4 retinas).
- No labeling of the cells with specific antibodies prior to FAC-sorting is needed.
